# Spatiotemporal Distribution of Harmful Algal Flora in the Tropical Estuarine Complex of Goa, India

**DOI:** 10.1100/2012/596276

**Published:** 2012-05-02

**Authors:** Suraksha M. Pednekar, S. G. Prabhu Matondkar, Vijaya Kerkar

**Affiliations:** ^1^Biological Oceanography Division, National Institute of Oceanography, Dona Paula, Goa 403004, India; ^2^Department of Botany, Goa University, Taleigao, Goa 403004, India

## Abstract

Mandovi and Zuari estuarine complex is monsoon-influenced estuaries located along the central west coast of India. During the past few years, there has been an increase in nutrient loading specially during monsoonal runoff which is responsible for the growth of harmful algal flora. To understand occurrence and distribution of harmful algal blooms species, daily/alternate day samplings were carried out in Mandovi and Zuari estuaries during 2007-2008 and 2008-2009 periods, respectively, comprising of monsoon (June–November) and nonmonsoon (December–May). In Mandovi, total 54 HAB species with 49 in monsoon and 36 during nonmonsoon period were reported. In Zuari, total 46 HAB species with 38 in monsoon and 41 were reported during nonmonsoon period. Bray-Curtis cluster analysis based on log-transformed phytoplankton density detected seven well-defined groups revealing spatiotemporal variability. The density of the dominant harmful algal species was significantly positively correlated with nutrients, but negatively correlated with salinity. The results of the study indicate that monsoon plays an important role in occurrence and distribution of harmful algal species having direct correlation with salinity variations and nutrient loading.

## 1. Introduction

Harmful algal blooms occur when the algal cells in the marine or fresh water grow out of proportion causing economic loss and severe impacts on marine life and human health [1]. The global study reveals that HAB-causing species are potentially harmful due to their physical structure, for example, several species of genus *Chaetoceros*, *Ceratium*, and *Prorocentrum* [2, 3]. Some are potentially toxic even at low concentrations (few cells per litre) and produce toxic effects affecting the marine life whose intern affects the human health.

In India, “red-tide” events were caused by *Noctiluca miliaris *along southern Kerala coast [4–6], and severe fish mortality was observed. Paralytic shellfish poisoning outbreak was observed in Mangalore along the west coast where the causative organism was not known [7, 8], and the stench of *Cochlodinium polykrikoides* was observed along the southern Malabar Coast by [9]. Many cysts of toxic dinoflagellate species have been reported in the sediments along the south west of India during the southwest monsoon period [10, 11]. “Red tide” event was caused by *Coscinodiscus centralis* on south west coast of India [12].

The occurrences of HAB species have been linked to a number of factors such as impact of weather conditions on water parameters like salinity, temperature, currents, nutrient concentrations, monsoonal pattern, and also the geomorphology of the location [13, 14]. Combination of some of these factors provides optimal conditions for HAB species to transform into bloom.

The Mandovi and Zuari are the most important estuaries of Goa along west coast of India and are typical tropical, tide-driven estuaries [15]. The estuaries differ in their geomorphology and rainfall pattern. The Mandovi estuary has wide mouth region and longer flushing period compared to Zuari [16]. The major source of pollution in the Zuari is the Mormugao port situated at Mormugao bay. In recent years, the anthropogenic activities like construction of jetties, ship building and boat traffic (cruising), mining activities, sewage discharge, agricultural runoff, and industrial effluents have increased in these estuaries [17–21]. This has lead to the increase in the macro- and micro-nutrients concentration specially nitrate and phosphate, suspended particulate matter, and trace elements influencing the algal blooms. Reports of harmful algal blooms have also been reported from Goa coast [22, 23]. However, no intensive study has been carried out on the HAB-forming species from these regions. Therefore the aim of the present investigation was (1) to study the spatiotemporal distribution of harmful algal species with respect to their occurrence and abundance and (2) the important environmental parameters that control these variations in the Mandovi and Zuari estuaries.

## 2. Materials and Methods

### 2.1. Study Site and Sampling

**Figure 1:**
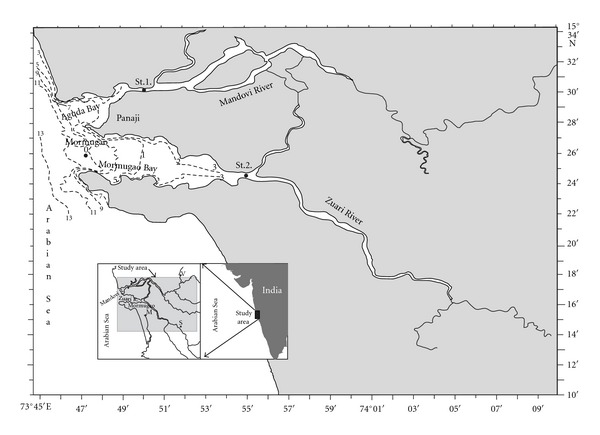
Map showing sampling stations (St.1-Mandovi and St.2-Zuari) estuaries of Goa.

The Mandovi (15°21′ and 15°31′N) and Zuari (73°45′ and 73°49′E) estuaries together with Cumbarjua canal form the major estuarine system of Goa. The rivers originate in Sahyadri hills in Western Ghats. Sampling in the Mandovi and Zuari River commenced at the start of the southwest monsoon during the years 2007 and 2008, respectively. Surface water samples were collected daily during monsoon (June–November) and alternate day during the nonmonsoon (December–May) from a single location and at fixed time (11:00 hrs) in both the estuaries. In the case of Mandovi, sampling station (St.1) was ~2 km upstream from the mouth of the estuary, and in Zuari, the site (St.2) was ~13 km upstream from the mouth of the estuary (Figure 1). These sites were chosen for two major reasons: first, because of its accessibility from the National Institute of Oceanography (NIO), vessel *CRV Sagar Shakti* which was to be anchored at the point during the entire southwest monsoon season, and second, on account of the large salinity range (0 to 37 psu) that this locations experienced [16]. At the highest high tide, the maximum depth at the sites was 6 m and 9.5 m in Mandovi and Zuari estuaries, respectively. Water samples were collected using a Niskin sampler attached to the Sea Bird CTD. The samples were then immediately transported under cold and dark conditions for further processing at the National Institute of Oceanography Laboratory.

### 2.2. Physicochemical Parameters

Rainfall over the entire west coast of India was obtained from the India Meteorological Department [24]. Salinity was measured with a “Salinometer” (Atago S/Mill, Japan, Salinity range 0~100 psu, resolution 1 psu between 10 and 20°C).

### 2.3. Nutrients Estimation

Nitrate (NO_3_), nitrite (NO_2_), phosphate (PO_4_), and silicate (SiO_4_) were analyzed using standard procedure [25].

### 2.4. Taxonomy of HAB Species

Samples for HAB species taxonomy and cell counts were collected in 500 mL opaque plastic bottles, fixed with a few drops of Lugol's iodine, preserved in 3% buffered formaldehyde, and stored under dark and cool conditions till further analysis. Samples were concentrated to 5–10 mL by carefully siphoning the top layer with a tube covered with a 10 *μ*m Nytex filter on one end. Sample concentrates were then carefully transferred to a 1 mL capacity Sedgwick-Rafter and counted. Phytoplankton cell identifications were based on standard taxonomic keys [26–28], and cell counts were carried out in duplicate.

### 2.5. Statistical Data Analysis

Data processing was done using PRIMER version 5.2.8 [29]. Bray-Curtis similarity index was constructed based on HAB density after log transformation. Following the division into groups from results of cluster analysis, the species having the greatest contribution to this division were determined using similarity percentage program (SIMPER). Principal component analysis was carried out using statistical package version 6.0. (StatSoft, Oklahoma USA). The purpose of the analysis is to see the effect of environmental variables on the abundance of dominant HABs species. The first principal component factor 1 accounts greater variability, and each succeeding factor explains the remaining variability possible in the data set. The ordination results for the first two most important factors (factor 1 and factor 2) were retained. Pearson's correlation was carried out in order to show the strength of association between the HAB species and environmental parameters.

## 3. Results

### 3.1. Physicochemical Parameters

#### 3.1.1. Rainfall

**Figure 2:**
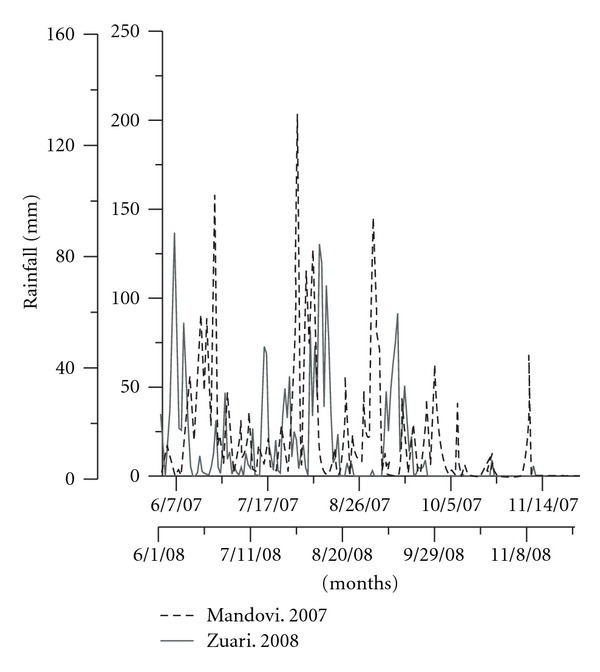
Rainfall pattern during the years 2007 and 2008.

The average rainfall during the years 2007-2008 varied between 2.52 mm and 35.91 mm (Figure 2) with highest value of 202.7 mm observed on 30th July, 2007. During 2008-2009, rainfall ranged from 0.35 mm to 27.16 mm with highest rainfall observed on the 8th June, 2008 (136.8 mm). In the year 2007, rainfall was above the normal value (3557.4 mm), while 2008 received below normal (2490.2 mm).

#### 3.1.2. Salinity

**Figure 3:**
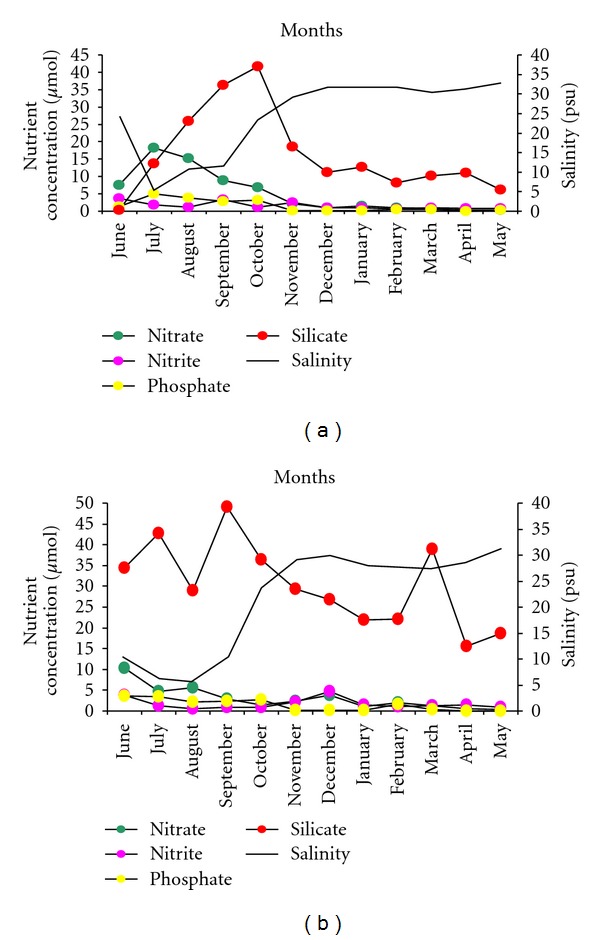
(a) Nutrients and salinity variations in Mandovi estuary. (b) Nutrients and salinity variations in Zuari estuary.

In Mandovi estuary, average salinity varied between 5 and 29 psu during monsoon with lowest in the month of July. The average salinity varied from 30 to 33 psu during nonmonsoon period (Figure 3(a)). During monsoon period in Zuari estuary, average salinity varied between 6 and 29 psu, and lowest salinity was recorded in August. In nonmonsoon period, average salinity varied between 27 and 31 psu (Figure 3(b)). 

#### 3.1.3. Nutrient Concentration

In Mandovi, the average highest nitrate of  18.21 *μ*mol was observed in July 2007, while in Zuari, nitrate was high (average 10.38 *μ*mol) in June 2008. During nonmonsoon period, there were no much variations in nitrate. Silicate concentration was on higher side throughout the study period in both the estuaries compared to nitrate, phosphate, and nitrite. In Mandovi, the highest average value of silicate was 41.61 *μ*mol (October 2007) and lowest of 0.25 *μ*mol in June 2007. Silicate concentration was high (49.12 *μ*mol) during September 2008 in the Zuari estuary. Nitrite and phosphate were higher during monsoon compared to nonmonsoon period (Figures 3(a) and 3(b)).

### 3.2. Spatiotemporal Distribution of HAB Species in Both the Estuaries

**Figure 4:**
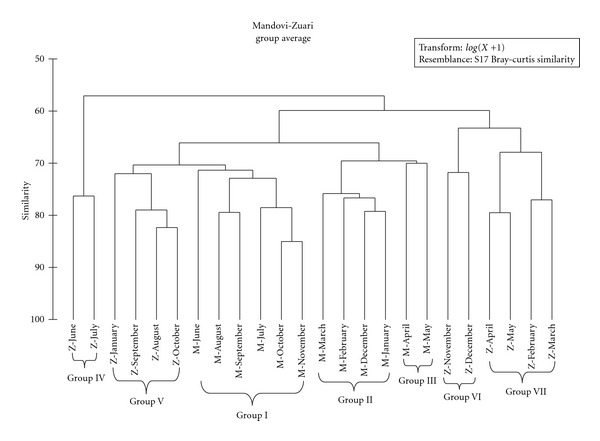
Dendrogram of cluster analysis based on Bray-Curtis similarity index in both the estuaries.

The list of phytoplankton species which are potentially harmful and toxic with their cell abundance in both the estuaries is given in Table 1. In Mandovi, total 54 HAB species with 49 in monsoon and 36 during nonmonsoon period were reported. In Zuari, total 46 HAB species with 38 in monsoon and 41 were reported during nonmonsoon period. The 54 HAB species in Mandovi comprised of 33 potentially harmful and 21 potentially toxic species. In Zuari, 46 total HAB species consisted of 26 potentially harmful and 20 potentially toxic species. The Bray-Curtis cluster analysis based on the log-transformed phytoplankton density detected seven well-defined groups revealing the spatiotemporal variability (Figure 4). Group I consisted of the monsoon samples of Mandovi estuary, while groups II (December–March) and III (April-May) consisted of the nonmonsoon months of Mandovi. Group IV consisted of the peak monsoon months (June-July) of Zuari, while group V consisted of the months of August–October, January. The nonmonsoon period was clustered into two groups, that is, group VI (November-December) and group VII (February–May). SIMPER analyses detected the species that contributed to the grouping and are summarized in Table 2.

### 3.3. Harmful Species Abundance and Environmental Variables

**Figure 5:**
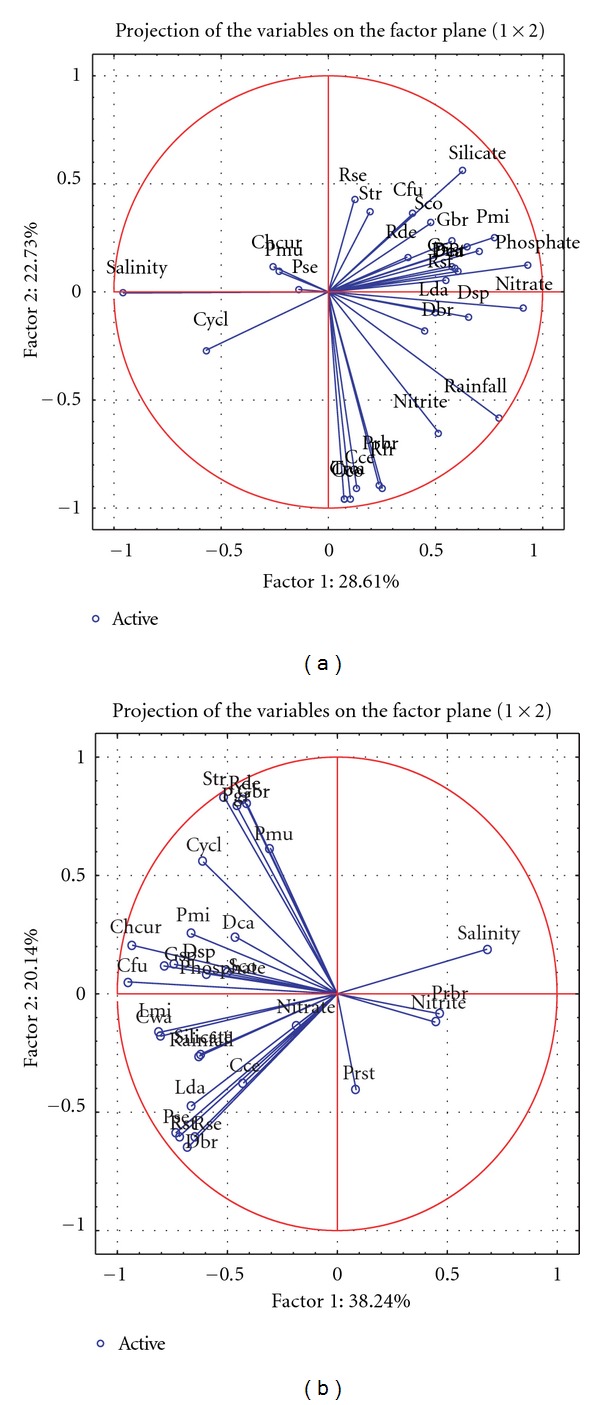
(a) PCA analysis of dominant harmful algal species abundance and physicochemical parameters in Mandovi estuary. Species acronyms: *Chaetoceros curvisetum-Chur, Coscinodiscus concinnus-Cco, Coscinodiscus wailesii-Cwa, Coscinodiscus centralis-Cce, Cylindrotheca closterium-Cycle, Ditylum brightwellii-Dbr, Leptocylindrus danicus-Lda, Leptocylindrus minimus-Lmi, Pseudo-nitzschia multiseriata-Pmu, Pseudo-nitzschia seriata-Pse, Rhizosolenia delicatula-Rde, Rhizosolenia fragilissima-Rfr, Rhizosolenia setigera-Rse, Rhizosolenia stolterforthii-Rst, Skeletonema costatum-Sco, Thalassiosira rotula-Tro, Ceratium furca-Cfu, Dinophysis caudata-Dca, Gymnodinium breve-Gbr, Gymnodinium splendens-Gsp, Prorocentrum gracile-Pgr, Prorocentrum micans-Pmi, Protoperidinium brevipes-Prbr, Protoperidinium steinii-Prst, Scrippsiella trochoidea-Str, and Distephanus speculum-Dsp. *(b) PCA analysis of dominant harmful algal species abundance and physicochemical parameters in Zuari estuary. Species acronyms: *Chaetoceros curvisetum-Chur, Coscinodiscus wailesii-Cwa, Coscinodiscus centralis-Cce, Cylindrotheca closterium-Cycl, Ditylum brightwellii-Dbr, Leptocylindrus danicus-Lda, Leptocylindrus minimus-Lmi, Pseudo-nitzschia multiseriata-Pmu, Pseudo-nitzschia seriata-Pse, Rhizosolenia delicatula-Rde, Rhizosolenia setigera-Rse, Rhizosolenia stolterforthii-Rst, Skeletonema costatum-Sco, Ceratium furca-Cfu, Dinophysis caudate-Dca, Gymnodinium breve-Gbr, Gymnodinium splendens-Gsp, Prorocentrum gracile-Pgr, Prorocentrum micans-Pmi, Protoperidinium brevipes-Prbr, Protoperidinium steinii-Prst, Scrippsiella trochoidea-Str, and Distephanus speculum-Dsp. *

PCA analysis was carried out using 26 dominant species in both the estuaries. In Mandovi (Figure 5(a)), the first two factors showed 50% of the variability with factor 1 showing 28.61%, and factor 2 contributed to 22.73% of the variance. HAB species like *Coscinodiscus concinnus, Coscinodiscus wailesii, Coscinodiscus centralis, Rhizosolenia fragilissima, and Thalassiosira rotula* showed significant positive correlation with nitrite and rainfall with *P* < 0.05. Species showing negative relation with salinity are *Ditylum brightwellii, Leptocylindrus danicus, Leptocylindrus minimus, Rhizosolenia stolterforthii, Rhizosolenia setigera, Skeletonema costatum, Ceratium furca, Gymnodinium breve, Gymnodinium splendens, Prorocentrum gracile, Scrippsiella trochoidea, Prorocentrum micans, Protoperidinium steinii,* and *Distephanus speculum P* < 0.05, while they showed positive relation with nutrients nitrate, phosphate, and silicate. *Pseudo-nitzschia multiseriata, Pseudo-nitzschia seriata, and Rhizosolenia delicatula* did not show significant relation with any of the environmental parameter. In Zuari estuary, the maximum variance of harmful species abundance with environmental parameters is explained by the first two factors (58%). Factor 1 explains 38.24% and factor 2 explains 20% (Figure 5(b)). *Chaetoceros curvisetum, Coscinodiscus wailesii, Ditylum brightwellii, Skeletonema costatum, Ceratium furca, Gymnodinium breve, *and* Prorocentrum gracile* showed significant positive correlation with phosphate (*P* < 0.05), silicate (*P* < 0.05), and rainfall (*P* < 0.05) but negative relation with salinity *P* < 0.05. *Coscinodiscus centralis, Leptocylindrus minimus, *and *Ceratium furca* are negatively related to nitrite (*P* < 0.05). *Protoperidinium brevipes* showed positive relation with salinity (*r* = 0.49; *P* < 0.05)*. Leptocylindrus minimus, Pseudo-nitzschia seriata, *and* Rhizosolenia stolterforthii* showed positive relation only with silicate (*P* < 0.05). *Protoperidinium steinii* was positively related only with phosphate (*r* = 0.3; *P* < 0.05). *Dinophysis caudata and Gymnodinium splendens* showed significant positive relation with rainfall *P* < 0.05.

## 4. Discussion

The present work provides a detailed study of the harmful algal bloom-forming species in two tropical estuaries, that is, Mandovi and Zuari along the central west coast of India. These estuarine systems receive large amount of runoff from June to September (south west monsoon); as a result, they are fresh water dominated during monsoon. Though partially landlocked, they are exposed to constant flushing and flooding, which considerably affect the environmental features of the estuaries. 

The study reveals the fact that daily/alternate day observations give more detailed account on the day-to-day changes in the phytoplankton biomass pattern especially on spatiotemporal distribution of harmful algal species with respect to the environmental parameters. Among the nutrients, nitrate was highest during monsoon in the Mandovi estuary, whereas silicate was high in the Zuari estuary throughout the study period. In comparison with earlier studies, an increase in the nutrient input [17–20, 30–34] is reported. The Mandovi-Zuari estuarine complex is the main transport route of the iron ore to the Mormugao harbour. In Mandovi, per year 5.21×10^6^ m^3^ of the sewage is disposed [35]. Land-based runoff was over 6004 Mm^3^ [36] during the monsoon wherein nutrients get transported to the estuaries leading to loading of the nutrients which is a major factor responsible for the growth of the HAB species [37–43]. 

The HAB species showed seasonality in the distribution as reflected in the Bray-Curtis similarity index which was purely based on the abundance of HAB species during each month. *Thalassiosira rotula, Rhizosolenia fragilissima,* and *Coscinodiscus concinnus* showed temporal variation and was positively related with nitrite and rainfall. *Coscinodiscus concinnus* was reported in Mahanadi estuary during postmonsoon and premonsoon periods due to their eurythermal and euryhaline nature and is known to grow quickly in estuarine conditions [44]. *Rhizosolenia delicatula* dominated in Mandovi estuary during the monsoon period. However, this species may occur anytime regardless of the monsoon season, but with varying cell densities. 

In the present investigation, potentially harmful pinnate diatom *Cylindrotheca closterium* was observed with high abundance in more saline waters during the nonmonsoon period in both the estuaries. The abundance was highest in Mandovi estuary during April-May (Table 2). Earlier this species was reported with high abundance in the Zuari estuary [45]. *Coscinodiscus centralis, Coscinodiscus wailesii, Protoperidinium brevipes, Protoperidinium steinii, Distephanus speculum, Ditylum brightwellii, Dinophysis caudata*, *Gymnodinium splendens, and Prorocentrum micans *showed strong positive relation with rainfall, nitrate, phosphate, and silicate (Figure 3(a)). These species are brought into the estuary during sea water influx from coastal water and are known to have preference for phosphate and silicate [40, 42]. During monsoon period, estuaries are rich in inorganic phosphate. In Zuari estuary, the source of phosphate is largely from the neritic waters, while in Mandovi, the high input is from the large number of tributaries [46]. Especially dinoflagellates like *Protoperidinium brevipes, Protoperidinium steinii, Dinophysis caudata*, *Gymnodinium splendens, and Prorocentrum micans* have diverse habitat and have ability to swim and sink under nutrient-stressed conditions [47, 48]. *Gymnodinium splendens* was reported for the first time during the monsoon phase in Mandovi. This species is known to proliferate in nutrient-enriched waters and also possesses the nutrient retrieval strategy: diel migration of nutrient-depleted dinoflagellate into nutrient-rich layers [49]. In general, dinoflagellates are known to grow best in environments that are rich not only in nitrogenous nutrients, but also in humic acid, fulvic acid, and other dissolved organic compounds that constitute the bulk of the colored dissolved organic matter (CDOM) pool [50–52]. One important source of CDOM is litter from the mangrove-laden banks upstream of the Mandovi River. During the monsoon, constant flooding and flushing of the mangrove beds causes leaching of CDOM from decaying litter. The bloom of *Protoperidinium* has been reported off Mangalore [53]. Silicoflagellate *Distephanus speculum* was observed but with low abundance and reported to cause depletion in oxygen concentration and produces harmful red tide [54]. 

The bloom of potentially harmful species *Ditylum brightwellii* observed in Zuari estuary during the monsoon period is a noteworthy as this species is known to occur in lower abundance in this estuary in other seasons [45]. The occurrence of *Leptocylindrus danicus, Leptocylindrus minimus, Rhizosolenia setigera, Rhizosolenia stolterforthii, Gymnodinium breve, Prorocentrum gracile, Scrippsiella trochoidea, Chaetoceros curvisetum, and Pseudo-nitzschia seriata* during the monsoon implies that they are typical monsoonal species which prefer low saline conditions. *Gymnodinium breve,* another toxic species, which showed spatial distribution with high abundance during monsoon period is of concern. This species can withstand shear/stress effects with autoregulated behavior which allows faster growth at lower light levels in waters enriched with nutrients [47, 49]. *Prorocentrum gracile and Scrippsiella trochoidea *were reported to grow in chemically disturbed (nutrient enriched through anthropogenic activities) marine waters [49]. Domoic acid-producing species of *Pseudo-nitzschia seriata* and *Pseudo-nitzschia multiseriata* have shown spatiotemporal variation in both the estuaries specially during the nonmonsoonal period when the nutrients are elevated but declining. Cultural experiments on these species showed that deficiency in silicate and phosphate triggers more domoic acid production [55]. 

*Ceratium furca *that showed spatiotemporal variability with high abundance during monsoon in Zuari estuary has the ability to take additional nutrient sources by mixotrophic feeding which might contribute to bloom formation and persistence allowing population growth even in low-nutrient conditions [56]. Potentially harmful species of *Ceratium furca* and *Skeletonema costatum* are found to be preponderant of these estuaries, euryhaline type, and grows in nutrient-enriched waters [45]. *Skeletonema costatum* is a cosmopolitan species found to grow at a wide range of salinity with high silicate concentration [45]. In this work, high abundance of this species was found in the Zuari estuary during the monsoon period. Bloom of *Skeletonema costatum* is also reported as indicator of pollution in the marine waters [44, 57]. Ammonia also plays important role in the growth of HAB species [38, 40]. Recent studies in Mandovi reported 1.79 *μ*mol of ammonia during the monsoon period [20]. Low salinity and high nutrients during the monsoon period favour the growth of HAB species in both the estuaries.

## 5. Conclusion

It can be concluded that monsoon acts as a major player in the spatiotemporal distribution of large number of harmful algal species. Abundance of HAB species is related to the increase in the nutrients in both the estuaries. The high level of nutrients is a result of increased anthropogenic activities in the two estuaries. Since fishery is an important activity in this region and during monsoon season when fishing in the coastal waters is banned, the major fishing takes place in these estuarine regions where shellfish dominates during monsoon period. Further, it can be concluded that increase in the nutrient can lead to harmful algal blooms during the monsoon which may be detrimental to marine life and humans. Reduction of nutrient loading by reducing the anthropogenic activities and to avoid extensive use of fertilizers for agricultural purpose is suggested.

## Figures and Tables

**Table 1:** List of potentially harmful and toxic species ranges of their cell counts *L* ^−1^ during monsoon and nonmonsoon period in Mandovi and Zuari estuaries of Goa.

Name of the estuary	Mandovi		Zuari	

Seasons	Monsoon (June 2007–November 2007)	Nonmonsoon (December 2007–May 2008)	Monsoon (June 2008–November 2008)	Monsoon (December 2008–May 2009)

Taxon	*n* = 186	*n* = 123	*n* = 163	*n* = 101
Diatom				
^+^ *Biddulphia mobiliensis*	1–8 (4 ± 3)	0–6 (1 ± 2)	0–40 (13 ± 20)	0–92 (51 ± 33)
^+^ *Biddulphia sinensis*	6–49 (22 ± 16)	1–246 (45 ± 99)	0–88 (39 ± 36)	0–62 (27 ± 24)
*Chaetoceros concavicornis*	0-1 (0 ± 1)	0–3 (1 ± 1)	0–273 (46 ± 111)	NP
^+++^ *Chaetoceros curvisetus*	26–161 (66 ± 55)	0–2793 (595 ± 1120)	0–1256 (624 ± 538)	0–144 (41 ± 65)
*Chaetoceros peruvianus*	0–6 (1 ± 2)	0–2 (0 ± 1)	NP	NP
*Chaetoceros sociale*	0–3 (1 ± 2)	NP	NP	NP
^+^ *Coscinodiscus centralis*	116–918 (299 ± 321)	32–103 (64 ± 31)	102–383 (204 ± 107)	86–381 (190 ± 115)
*Coscinodiscus concinnus*	0–77 (13 ± 31)	NP	NP	NP
*Coscinodiscus wailesii*	6–1860 (352 ± 741)	9–36 (20 ± 10)	173–1016 (580 ± 357)	51–261 (116 ± 74)
^+,++^ *Cylindrotheca closterium*	180–1059 (429 ± 331)	674–2273 (1512 ± 693)	88–5326 (1566 ± 2047)	145–1657 (821 ± 624)
^+,++^ *Ditylum brightwellii*	16–294 (93 ± 110)	0–63 (27 ± 27)	374–13014 (3484 ± 4831)	49–2134 (461 ± 823)
*^+^Eucampia zodiacus *	0–7 (3 ± 2)	0–2 (1 ± 1)	0–115 (26 ± 47)	0–48 (13 ± 21)
^+,++^ *Leptocylindrus danicus*	15–790 (235 ± 286)	3–659 (133 ± 259)	28–7521 (1716 ± 2908)	30–2356 (802 ± 1085)
*^+^Leptocylindrus minimus*	1–163 (53 ± 62)	0–92 (21 ± 36)	0–2511 (765 ± 1010)	0–545 (287 ± 195)
*Pseudo-nitzschia australis	NP	NP	NP	0–475 (126 ± 204)
^++,∗^ *Pseudo-nitzschia multiseriata*	0–9 (3 ± 3)	0–142 (32 ± 56)	0–2560 (477 ± 1023)	0–2112 (373 ± 825)
*Pseudo-nitzschia pungens	0–6 (3 ± 2)	0–11 (3 ± 4)	0–38 (6 ± 16)	NP
*Pseudo-nitzschia pseudodelicatissima	NP	NP	NP	0–5760 (960 ± 2352)
^+,∗^ *Pseudo-nitzschia seriata*	5–70 (32 ± 23)	0–315 (102 ± 142)	0–4080 (869 ± 1616)	77–266 (138 ± 73)
^+^ *Rhizosolenia alata*	0–2 (1 ± 1)	0–3 (1 ± 1)	0–42 (7 ± 17)	NP
^+^ *Rhizosolenia delicatula *	3–307 (88 ± 120)	0–176 (37 ± 69)	0–560 (162 ± 227)	NP
*Rhizosolenia fragilissima*	0–201 (35 ± 81)	NP	NP	NP
^+,++^ *Rhizosolenia setigera*	3–1242 (233 ± 496)	6–261 (133 ± 259)	39–10383 (2114 ± 4072)	101–2604 (822 ± 1109)
^+,++^ *Rhizosolenia stolterforthii*	0–378 (95 ± 155)	0–4 (1 ± 2)	0–12166 (2572 ± 4759)	0–115 (38 ± 59)
^+,++^ *Skeletonema costatum*	17–3421 (1396 ± 1374)	19–2347 (518 ± 904)	433–12348 (7162 ± 4411)	32–13246 (2619 ± 5228)
*Thalassiosira rotula*	0–10 (2 ± 4)	NP	NP	NP
^+^ *Thalassiosira subtilis*	NP	NP	0–28 (5 ± 11)	0–5790 (1055 ± 2330)

Dinoflagellate				
*Alexandrium catenella	0-1 (0 ± 0)	NP	0–224 (51 ± 87)	0–288 (58 ± 115)
*Alexandrium tamarense	0–2 (0 ± 1)	NP	NP	0–5104 (851 ± 2084)
*Alexandrium ostenfeldii	1–32 (11 ± 13)	0–12 (2 ± 5)	NP	0–54 (9 ± 22)
^++,+++^ *Ceratium furca*	31–842 (215 ± 311)	14–122 (49 ± 40)	67–2635 (1298 ± 1111)	75–213 (122 ± 48)
^+^ *Ceratium fusus*	NP	NP	NP	0–40 (16 ± 18)
*Dinophysis acuta	NP	NP	NP	0–88 (23 ± 37)
*Dinophysis acuminata	0-1 (0 ± 0)	NP	NP	0–130 (22 ± 53)
^+,∗^ *Dinophysis caudata*	0–48 (12 ± 19)	0–2 (1 ± 1)	0–1108 (196 ± 448)	NP
*Dinophysis fortii	0–10 (2 ± 4)	NP	NP	NP
*Dinophysis miles	NP	NP	NP	0–96 (23 ± 40)
^+,∗^ *Dinophysis mitra*	0-1 (0 ± 0)	NP	NP	0–112 (45 ± 54)
*Gonyaulax polygramma*	NP	0–15 (5 ± 6)	NP	NP
^+++,∗^ *Gymnodinium breve*	0–381 (76 ± 150)	0–2 (0 ± 1)	0–5291 (952 ± 2131)	NP
*Gymnodinium mikimotoi	NP	0–2 (0 ± 1)	NP	NP
^++,∗^ *Gymnodinium splendens*	1–1122 (265 ± 453)	0–2 (1 ± 1)	0–261 (110 ± 91)	0–82 (38 ± 33)
*Gyrodinium spirale*	0–186 (35 ± 74)	0–6 (3 ± 2)	0–309 (96 ± 125)	0–68 (22 ± 33)
^+^ *Noctiluca scintillans*	0–2 (0 ± 1)	NP	0–128 (22 ± 16)	NP
*Prorocentrum cordatum	1–6 (3 ± 2)	0–10 (4 ± 5)	0–42 (14 ± 21)	NP
*Prorocentrum dentatum	0–2 (0 ± 1)	NP	NP	NP
*Prorocentrum gracile*	1–132 (43 ± 49)	9–23 (16 ± 6)	0–1646 (346 ± 644)	0–80 (44 ± 28)
^+^ *Prorocentrum micans*	26–399 (165 ± 137)	23–82 (45 ± 21)	36–3091 (800 ± 1179)	68–142 (97 ± 28)
^+,∗^ *Protoperidinium depressum*	2–38 (14 ± 14)	3–14 (9 ± 5)	0–119 (52 ± 46)	0–82 (14 ± 33)
*Protoperidinium brevipes	0–31 (9 ± 14)	NP	0–34 (6 ± 14)	0–156 (60 ± 64)
*Protoperidinium steinii	0–117 (35 ± 55)	NP	0–410 (120 ± 189)	68–549 (192 ± 183)
*Scrippsiella trochoidea*	2–1212 (316 ± 498)	0–29 (15 ± 10)	0–4260 (1025 ± 1717)	0–734 (171 ± 279)

Other Algae				
*Dictyocha fibula*	1–45 (16 ± 17)	0–25 (9 ± 9)	0–101 (23 ± 41)	0–143 (44 ± 57)
*Distephanus speculum*	0–38 (9 ± 15)	0–3 (1 ± 1)	0–859 (228 ± 336)	6–49 (22 ± 16)
*Microcystis aeruginosa	0-1 (0 ± 1)	NP	NP	NP
^+++,∗^ *Trichodesmium erythraeum*	2–381 (69 ± 153)	1–14 (8 ± 5)	30–272 (96 ± 93)	0–155 (43 ± 60)

*n*: no. of days.

NP: not present.

*Potentially toxic species.

^+^Reported during 1980-1981.

^++^Bloom now.

^+++^Bloom during 1980-1981.

**Table 2:** Percent compositions of dominant HAB species in each group outlined by the cluster analysis based on SIMPER analysis.

Genera/species							
	Different cluster groups						
	Group I	Group II	Group III	Group IV	Group V	Group VI	Group VII
	Total HAB composition (%)						
*Chaetoceros curvisetum*	4.82	—	—	7.73	5.20	—	—
*Coscinodiscus concinnus*	7.88	—	—	—	—	—	—
*Coscinodiscus wailesii*	3.71	6.44	7.53	10.80	5.47	6.00	6.95
*Coscinodiscus centralis*	4.48	7.25	8.12	9.22	4.87	6.77	7.42
*Cylindrotheca closterium*	5.19	11.27	14.74	8.93	6.75	7.47	8.11
*Ditylum brightwellii*	3.47	6.38	—	15.94	6.14	8.46	6.59
*Leptocylindrus danicus*	4.28	6.94	8.46	6.69	6.20	8.09	5.75
*Leptocylindrus minimus*	4.18	3.47	6.87	—	5.84	—	8.32
*Pseudo-nitzschia multiseriata*	—	7.09	—	—	4.99	—	—
*Pseudo-nitzschia seriata*	3.41	3.40	7.69	—	4.48	6.60	6.88
*Rhizosolenia delicatula*	4.30	—	—	—	—	—	—
*Rhizosolenia fragilissima*	6.39	—	—	—	—	—	—
*Rhizosolenia setigera*	3.48	7.68	9.03	7.33	6.16	7.48	7.35
*Rhizosolenia stolterforthii*	1.98	—	—	—	4.98	7.22	—
*Skeletonema costatum*	5.60	8.29	8.99	17.78	8.06	9.07	7.06
*Thalassiosira rotula*	6.34	—	—	—	—	—	—
*Ceratium furca*	4.54	7.00	6.79	8.40	5.56	7.40	7.08
*Dinophysis caudata*	—	—	6.27	—	—	—	—
*Gymnodinium breve*	3.86	—	—	—	2.01	—	—
*Gymnodinium splendens*	3.41	—	—	—	4.29	6.03	—
*Prorocentrum gracile*	3.58	5.89	6.74	—	4.14	—	5.66
*Prorocentrum micans*	4.48	6.71	8.76	7.17	5.34	6.02	6.86
*Protoperidinium brevipes*	6.38	—	—	—	—	—	5.94
*Protoperidinium steinii*	—	—	—	—	—	7.17	6.85
*Scrippsiella trochoidea*	4.23	6.98	—	—	5.21	6.24	3.17
*Distephanus speculum*	—	5.23	—	—	4.29	—	—
